# A model of integrated remote monitoring and behaviour change for osteoarthritis

**DOI:** 10.1186/s12891-021-04555-4

**Published:** 2021-08-09

**Authors:** Christopher Tack

**Affiliations:** Guy’s and St Thomas’ NHS Foundation Trust, Great Maze Pond, London, SE1 9RT UK

**Keywords:** Digital behaviour change interventions, Remote monitoring, Osteoarthritis, Persuasive design, Mobile health

## Abstract

**Background:**

The National Institute for Health and Care Excellence recommends the use of digital and mobile health technologies to facilitate behaviour change interventions. Due to its high prevalence and dependence upon patient self-management strategies, osteoarthritis is one musculoskeletal condition which may benefit from such approaches. This is particularly pertinent due to the increasing use of remote monitoring technologies to collect patient data and facilitate self-management in individuals outside of hospital clinics. In practice however, application of digital behaviour change interventions is difficult due to insufficient reporting of behaviour change theories in the current literature. When digital technologies are employed to alter behaviour change in osteoarthritis, they often focus on physical activity. Currently, such interventions focus of self-efficacy but do not often explicitly report the behaviour change techniques they use to facilitate these changes.

**Methods:**

This paper proposes a new model of integrating specific behaviour change principles (persuasive design) in an integrated model of remote monitoring and digital behaviour change interventions for osteoarthritis.

**Results:**

There is potential to combine remote monitoring systems of patient data through digital and mobile technologies with behaviour change principles to improve physical activity behaviours in individuals with osteoarthritis. The use of persuasive design principles (e.g. prompts or nudges) through mobile notifications and strategic system design can be directed to enhance behaviour change. A validated measure of behaviour change, such as the patient activation measure, will allow effective evaluation of such systems.

**Conclusions:**

Digital behaviour change interventions should be directed towards the underlying principles of behaviour change they employ, although this is not commonly reported in practice. Such interventions can be integrated within remote monitoring pathways using persuasive design techniques to enhance patient activation. This approach can enhance self-management in individuals with musculoskeletal conditions, such as osteoarthritis.

## Background

In October 2020 the National Institute for Health and Care Excellence (NICE) published national guidance in the UK on digital and mobile health intervention for behaviour change [1]. This guidance recommends the use of digital technology as an option for behaviour change interventions both independently from clinicians, or in order to deliver interventions remotely. The guideline covers how digital behaviour change interventions (DBCIs), can support the adoption of health behaviours related to diet, smoking cessation, safe sex, alcohol consumption and physical activity. In order to supplement behaviour change, the guideline advises the use of interventions that include self-monitoring through activity trackers or e-diaries to guide progression towards a patient’s goals. Whilst the evidence collated shows variability in clinical outcomes, the use of DBCIs to facilitate greater physical activity is recommended.

Remote monitoring (RM) involves the use of digital tele-monitoring technology to measure patient data in environments away from clinical sites through a mechanism allowing remote observation of their clinical status by clinicians [2]. In response to the coronavirus pandemic the UK encouraged the adoption of RM solutions to allow care to be provided to patients in their own homes [3]. Devices (such as computers, smart phones, tablets) and software (e.g. mobile applications) provide opportunities to measure metrics such as exercise or medicine adherence, or physical data (such as blood pressure or blood glucose) at home. The data is then transferred to the healthcare providers for monitoring, analysis and as required to initiate a therapeutic intervention [4]. Whilst there is evidence for RM significantly improving quality of life, enhancing patient independence and assisting clinicians to proactively manage a person’s care [5]; there is less evidence of how DBCIs can integrate with RM pathways. The use of RM as a behaviour change technique, within a framework of a selected behaviour change theory, has not been reported, and variability exists regarding the reactive interventions which are provided in response to RM. Despite this, RM systems are attributed all the potential outcomes associated with the care it elicits, but it is unclear how RM attains these outcomes. By better defining how DBCIs are employed as part of RM pathways, clinicians and services can delineate which elements of the digital ecosystem best benefit their patients. Examples of wearable technology sensors for remote monitoring of musculoskeletal disorders, integrated with behavioural change principles remain experimental [6]. It is the purpose of this commentary to propose a model of integrated remote monitoring and digital behaviour interventions for one specific musculoskeletal condition-osteoarthritis. Further the model shall define how remote monitoring may be enhanced through integration of digital behaviour change principles.

### Behaviour change theory

Behaviour change interventions (BCIs) are defined as a coordinated set of activities designed to modify specific behaviours [7]. They should be grounded in conceptual theories of behaviour, that is the psychological processes hypothesised to regulate behaviour change (e.g. self-efficacy or planned behaviour). Due consideration of the psychological concepts to which they are directed (e.g. intent formation or relapse prevention) should be taken [8]. BCIs should be directed to a target individual or population, and with due respect to the proposed actions they cause and subsequent outcomes [9]. The Theoretical Domains Framework (TDF) was developed to simplify behaviour change theory to assist evidence based implementation [10]. 33 theories are clustered into 14 domains, which have been mapped to the well-established Behaviour Change Wheel model [11, 12]. This model synthesises 19 frameworks of behaviour change around three components: Capability, Opportunity and Motivation to Behaviour (COM-B). COM-B helps identify which TDF domains are most likely to influence behaviour change, and in combination these models support BCI developers in ensuring interventions incorporate behaviour change theory to increase the likely success of changing behaviour [13]. Michie et al. [11] also have devised a taxonomy of behaviour change techniques, which are the smallest active components of BCIs which we expect could change behaviour. Models of BCIs must consider all various components: psychological theories of behaviour change, underpinning behaviour change theories, and the most effective behaviour change techniques required to meet their aims. There is of course, interplay between these categories (e.g. goal setting is considered a theory and a technique in some literature).

DBCIs are health interventions (delivered through digital technology) which aim to facilitate change in behaviour. Taj et al. [14] found, in a scoping review of the last 20 years of DBCI research, commonalities in which theories and techniques were employed. Unfortunately, most studies (approximately 67%) do not report the theories or techniques which they employ. The most common theory reported was social cognitive theory (29%) followed by the transtheoretical model (10%). Frameworks, such as persuasive system design (19%) or gamification (17%) were equally underreported. Most commonly used techniques were goal setting (36%) and self-monitoring (33%). However, the focus on goal-directed self-monitoring to enhance self-management, is not evidently supported by behavioural facilitation through cues, prompts, reminders or reinforcement, as this approach was rarely described across studies [14]. This is despite the most common platform used being mobile phones (rather than SMS or the Web) where notification technology can be used. This is contradictory to recommendations supporting the need for clear links between DBCIs and theoretical mechanisms of change [13], and strategic integration of behaviour change techniques into digital interventions.

One model, found in only 20% of studies [14], is the Fogg behavioural model of persuasion (FBM). Fogg [15] states that in order for a behaviour to occur three elements should be present: motivation, ability and prompt (or trigger). The latter component is a cue or call to action which facilitates a change in behaviour (e.g. an alarm or a well-timed piece of feedback). This model has similarities with another rarely considered behavioural theory in DBCI development- nudge theory [16]. Nudges aim to alter people’s behaviour through the presentation of positive behavioural options, without forbidding any other option within their architecture of decision making [17]. Applied to a DBCI, this could be auto-enrolment with a programme of education shared daily via mobile app notification, sending an activity reminder SMS, or eliciting implementation intentions by framing a question- e.g. “Do you plan to exercise today?”. Nudges are easy and cheap to deploy, and whilst presenting an option for behaviour, still allow individuals to make decisions [18]. As such, nudges can be considered a type of prompt as described in Fogg’s model, as both are synonymous with the provision of a timely, appropriate response to a patient’s input into the system (e.g. reduce adherence or low reported score of patient reported outcome measures). Within DBCIs these theories may provide strategies by which patient behaviour can be positively directed, and the integration with remote monitoring technology provides opportunities to be responsive to the patient’s current state and actions. However to date, the implementation of such theories has been limited largely by the misalignment of persuasive design principles and the theoretical basis of interventions [19]. Oinas-Kukkonen and Harjumaa [20] elaborate upon the Fogg triad to align these principles with software requirements and to produce design guidelines for persuasive system design. This includes re-categorisation of persuasive design principles to primary task, dialogue, system credibility and social support. Further, they provide examples of implementations to guide users. Similarly, the Behavioural Intervention Technology Model provides a framework to integrate clinical aims of behaviour change within the technological architecture of the intervention [21]. Thus, providing the thread between behavioural theory, through clinical context and the specificities of an intervention, towards a distinct workflow to define when the intervention will be delivered. These additional frameworks should guide practical implementation in the future. BCIs are shown to assist individuals and populations to make lifestyle behaviour changes towards achieving better health, reductions in healthcare utilisation and expenditure [14]. The implementation of BCIs into digital technology have equal potential to show such benefits [22], particularly using mobile technology to collect patient metrics remotely and to provide “just-in-time” interventions to support change [23]. This is the basis by which the proposed model of integrated remote monitoring and digital behaviour change for osteoarthritis will be based.

### DBCIs for behaviour change in individuals with osteoarthritis

Physical activity and self-management are core components of treatment for osteoarthritis (OA) [24, 25], and DBCIs provide the opportunity to promote health in a cost effective and accessible manner to provide information and encouragement to enhance activity [26]. It is evident that technology offers simple and effective means to facilitate self-management of health through monitoring of physical activity and subsequent behaviour change [27]. Advancements in wearable technology (e.g. Fitbit, Apple iWatch, etc.) provide a progression from the effectiveness of smartphone pedometers [28, 29] to facilitate common behaviour techniques such as goal setting, self-monitoring and action-planning [30]. Mobile and wearable technologies can also facilitate improvements in adherence to treatment plans for physical activity or medication [31]. The principle behaviour change technique used to this end is enhancing knowledge toward greater self-efficacy, rather than self-monitoring or feedback responses [32]. This demonstrates the opportunity to have more sophisticated integration of behaviour change theory into technology supporting remote monitoring and DBCIs [29].

Berry et al. [33]) examined nine studies on the use of DBCIs to facilitate physical activity in OA. Across these studies, six interventions showed a statistically significant benefit on increasing physical activity [34–40]. The interventions described included web-based self-guided physical activity intervention [34–45], a self-guided smartphone app with a wearable monitor [36], and two online programmes with clinician guided interactive support [37, 39]. The digital programmes showed increases in physical activity for up to one year post intervention, but with no greater impact in those providing human-guided support. The most commonly employed theory of change was social cognitive theory or self-efficacy, and the most used behaviour change techniques are goal setting, action planning, feedback provision and self-monitoring.

Lorig, Ritter, Laurent et al. [37] describe the results of a randomised controlled trial examining the effect of the interactive, online Arthritis self-management program (ASMP) on health status, self-efficacy and healthcare utilisation. This DBCI used exercise logs, medication diaries, a tailored exercise program, and e-learning resources; taught interactively to enhance self-efficacy. Whilst moderators assisted participants in using the platform, they did not deliver content nor respond strategically to participants during the course. The results indicate that the programme was effective in increasing minutes per week of aerobic exercise (22.6 +/-SD 100.6 vs 0.316 +/-SD 100.3) and self-efficacy on a 10 point scale (0.801 +/-SD 2.17 vs 0.259 +/-SD 1.79), however was less effective in increasing minutes per week of stretching and strengthening exercise (6.29 +/-SD 55.8 vs 8.26 +/-SD 58.0). This indicates that this DBCI partially improves behaviour towards positive health outcomes, but poses the question whether more strategic application of behaviour change theory and techniques may optimise this impact.

A wide range of behaviour change techniques can enhance physical activity adherence in chronic musculoskeletal conditions (including OA), and that the combination of techniques leads to better outcomes [41]. Lambert et al. [42] describe the effects of a mobile app-based exercise program with remote support via telephone and SMS message on adherence in individuals with musculoskeletal conditions (compared to paper-based exercise resources). Importantly, the clinical team responded to poor adherence by contacting the participants to ensure they were not experiencing issues and to encourage them to perform their exercises. They also provided weekly motivation messages to all participants in the intervention group. The findings showed that the intervention group showed a statistically significant between-group difference for self-reported adherence (1.3/ 11 points, 95% CI 0.2-0.23; p= 0.01). They also showed a statistically significant between-group difference for the patient-specific functional score for the intervention group (0.9/ 11 points, 95% CI 0.1-1.7). However, there was no statistically significant effect seen for World Health Organisation Disability Assessment Schedule, patient satisfaction, or assessor-reported adherence. Whilst the authors do not explicitly describe the behaviour change theory being employed, this is an example of a DBCI implemented with a feedback system responsive to patient metrics collected through remote monitoring.

### A model of integrated remote monitoring and DBCI

It is evident that the wide provision of remote monitoring services can be a system to facilitate engagement with physical activity and self-management in individuals with OA. Strategic implementation of behaviour change principles into digital interventions can enhance their effectiveness to optimise self-efficacy and adherence to physical activity. The digital technology used for remote monitoring (e.g. mobile apps, wearable devices), and the ability to observe and respond to patient data both synchronously and asynchronously, is an ideal platform for persuasive system design. As with many DBCIs, behaviour change techniques often overlap with persuasive design principles, such as monitoring options, prompts/cues, rewards. The model proposed here incorporates the following key elements:

- Remote monitoring through an online/ mobile app platform (including wearable technology with real time data collection of physical activity or self-reported adherence)
- Personalised information and digital education resources
- Clinician alerts for reduced adherence
- Automated feedback, praise and reward systems in response to positive behaviours (using notification technologies)
- Incorporation of persuasive design principles in planned programmes of patient specific feedback (clinician guided and automated)

**Fig. 1:**
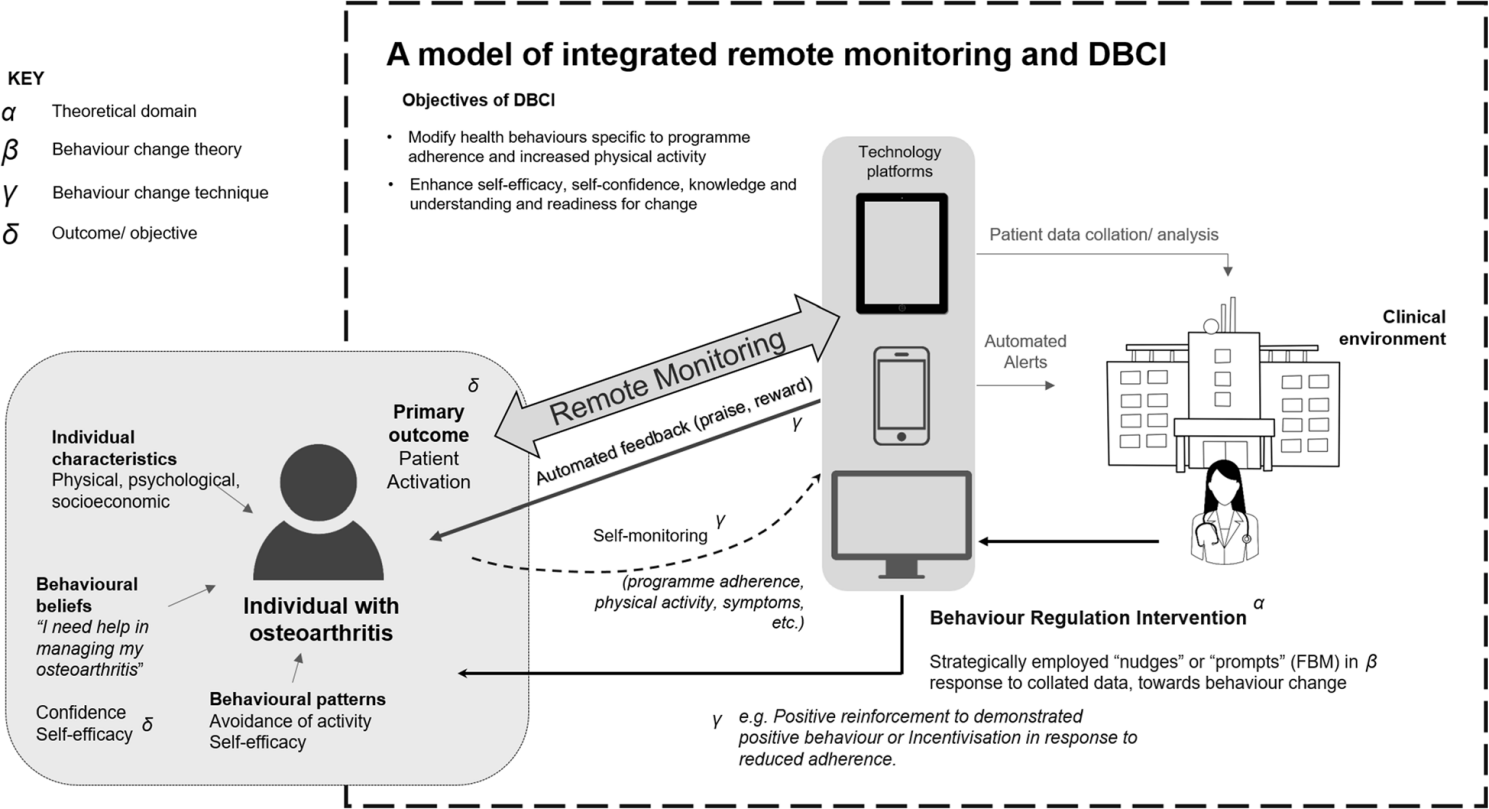
An integrated model of remote monitoring and DBCI. A simplified illustration is provided to present the link between the psychological theoretical domain, behaviour change theory and selected behaviour change techniques within the interconnected remote monitoring and DBCI model

Figure 1 provides a simple illustration of the model. Nudging is a concept of user assistance in digital environments [43] based on social-psychological and cognitive theories of human decision making [16]. Nudges have been used to positively influence several health behaviours relevant to OA management, including increasing physical activity [44] and healthy eating [45]. Mollenkamp et al. [46] describe a systematic review of the effectiveness of nudges in improving self-management for chronic diseases. Their findings indicate that the available evidence, although sparse, shows nudging can improve self-management of chronic diseases. They concur that reminders, feedback and planning prompts are the most useful techniques to facilitate better self-management. In the example of a mobile app to guide self-management for OA, this may include subtle and graduated exposure to ideas which challenge specific beliefs, customised educational information, praise or reward notifications in response to self-reporting of good behaviour, reminders to perform healthy behaviours, or warnings in response to negative behaviour (e.g. reduced adherence). Whether such responses are considered “nudges” or “prompts” will depend upon the degree by which responses to patients are built within an open decision architecture allowing choice, rather than being compulsory or didactive. Either through direct prompts as per Fogg’s model or through modifying the patient’s decision architecture through nudging, the ability to direct behaviour change should be incorporated into DBCIs.

### Patient activation

A persisting question, is to which outcome integrated remote monitoring and DBCIs should be directed. It is recommended that validated measures should be incorporated into such tools to provide a baseline of readiness for change [47]. For OA, such a measure could be the Patient Activation Measure (PAM). The PAM is a 13-item scale designed to measure an individual’s knowledge, skill and confidence in managing their health, and hence their self-management capabilities [48]. Importantly, the construct of activation is not condition specific, nor focused on a specific behaviour, and therefore is broader than singular behaviour change concepts (e.g. readiness for change, self-efficacy). Rather, the PAM assess various facets of the individual (e.g. patient knowledge, confidence, beliefs) and predicts a range of behaviours (e.g. exercise behaviours or disease-specific behaviours) [49]. Increases in levels of activation are associated with subsequent improvements in outcomes [49–51], and more positive health behaviours [49]. The PAM is considered both reliable and valid in its use to measure activation in individuals with osteoarthritis [52].

The use of the PAM as a measure within an integrated model of remote monitoring and behaviour change is supported by previous research. Edbrooke-Childs et al. [53] report a feasibility study looking at a mobile app interface to improve patient activation for mental health. They found the app an acceptable method to measure activation in young people attending child mental health services. The use of a wireless self-monitoring program via smartphone and online platform to improve patient activation towards enhancing blood pressure control in hypertensive patients (n=95) was evaluated in a randomised controlled trial [54]. Measures included patient activation, blood pressure control and health behaviours (e.g. smoking cessation). In comparison to a standard management program, the digital self-monitoring programme saw that relative improvements in activation were associated with increases in blood pressure control (p=<0.02), reducing cigarette smoking (p<0.001), and systolic (p=<0.27)/ diastolic blood pressure (p=<0.007). This is despite no significant change in mean PAM scores across all participants. These early studies show the potential for the PAM to be incorporated in digital intervention and measurement systems.

### Future development

The use of remote monitoring as a tool of patient-led self-monitoring towards behaviour regulation, can be optimised through the strategic deployment of nudges and prompts. It is proposed this may enhance the effectiveness of the DBCI and integrates behaviour change theory into practice. The theoretical model of integrated remote monitoring and behaviour change incorporating persuasive design evidently requires specific future research to assess its effectiveness. It is essential that studies should explicitly state the combination of behavioural theories and techniques being employed in the intervention studied, in order to gain understanding of the causative mechanisms behind change in the PAM and subsequent behaviours [13]. The dosage of prompts/ nudges should be described to determine the most effective pattern and frequency by which behaviour can be optimised.

In order to optimise clinical utility of DBCIs, a user-centred design approach should be adopted, and acceptability and feasibility testing with users should be standard practice. This may ensure barriers to adoption of technologies, such as access to technology or decline in technology usage over time, can be addressed [55]. User-centred design may also allow personalisation of digital interfaces to members of the target population, enhancing desirability and perhaps their success [56]. It may also identify where nudge-based persuasion techniques may be considered manipulative and unacceptable for users, to ensure interventions remain uncontroversial and accepted. Personalisation of the DBCI may also ensure barriers to engagement with individuals with self-reported lower levels of health literacy are addressed. Whilst health literacy is found to not predict technology use, it is associated with certain activities [57]. For example, those with low self-report health literacy are found less likely to use search engines, and preferred mobile apps (p=0.026) or SMS (p=0.013) to receive information [57]. This demonstrates how DBCIs can be designed to overcome this issue. Similarly, co-design with users will assist DBCIs to be developed with due consideration of the varied ethical issues related to data ownership and sharing. Challenges associated with data collection through mobile devices include (but are not limited to) privacy protection, minimisation of third party data disclosure and use, and governance and evaluation [58]. Future success of the model will rely on user engagement around these issues.

## Conclusions

DBCIs commonly are implemented without due reporting of the underlying principles of behaviour change they employ. Integration of DBCIs with remote monitoring pathways provide the opportunity to use persuasive design techniques to enhance patient activation and subsequent behaviour change. The enhancement of self-management in individuals with OA is one example of a musculoskeletal disorder where this model could show promise, and future research should consider how nudges/ prompts could be strategically used to enhance patient activation and changes in physical activity.

## Data Availability

Not applicable

## Abbreviations

ASMP: Arthritis self-management program
BCI: Behaviour Change Intervention
COM-B: Capability, Opportunity and Motivation to Behaviour
DCBI: Digital Behaviour Change Intervention
NICE: National Institute for Health and Care Excellence
OA: Osteoarthritis
PAM: Patient Activation Measure
RM: Remote monitoring
TDF: Theoretical Domains Framework
